# *Lampe1*: An ENU-Germline Mutation Causing Spontaneous Hepatosteatosis Identified through Targeted Exon-Enrichment and Next-Generation Sequencing

**DOI:** 10.1371/journal.pone.0021979

**Published:** 2011-07-07

**Authors:** Rachel Sheridan, Kristin Lampe, Shiva Kumar Shanmukhappa, Patrick Putnam, Mehdi Keddache, Senad Divanovic, Jorge Bezerra, Kasper Hoebe

**Affiliations:** 1 Department of Pathology and Laboratory Medicine, Cincinnati Children's Hospital Medical Center, Cincinnati, Ohio, United States of America; 2 Department of Molecular Immunology, Cincinnati Children's Hospital Research Foundation, Cincinnati, Ohio, United States of America; 3 Division of Comparative Medicine and Pathology, New England Primate Research Center, Harvard Medical School, Southborough, Massachusetts, United States of America; 4 Department of Human Genetics, Cincinnati Children's Hospital Research Foundation, Cincinnati, Ohio, United States of America; 5 Department of Gastroenterology, Hepatology and Nutrition, Cincinnati Children's Hospital Research Foundation, Cincinnati, Ohio, United States of America; Duke University Medical Center, United States of America

## Abstract

Using a small scale ENU mutagenesis approach we identified a recessive germline mutant, designated *Lampe1* that exhibited growth retardation and spontaneous hepatosteatosis. Low resolution mapping based on 20 intercrossed *Lampe1* mice revealed linkage to a ∼14 Mb interval on the distal site of chromosome 11 containing a total of 285 genes. Exons and 50 bp flanking sequences within the critical region were enriched with sequence capture microarrays and subsequently analyzed by next-generation sequencing. Using this approach 98.1 percent of the targeted DNA was covered with a depth of 10 or more reads per nucleotide and 3 homozygote mutations were identified. Two mutations represented intronic nucleotide changes whereas one mutation affected a splice donor site in intron 11–12 of Palmitoyl Acetyl-coenzyme A oxygenase-1 (*Acox1*), causing skipping of exon 12. Phenotyping of *Acox1^Lampe1^* mutants revealed a progression from hepatosteatosis to steatohepatitis, and ultimately hepatocellular carcinoma. The current approach provides a highly efficient and affordable method to identify causative mutations induced by ENU mutagenesis and animal models relevant to human pathology.

## Introduction

Forward genetics has made a significant impact in identifying genes involved in the host response to pathogens, or that are involved in the development of spontaneous disease [1]. Recently, the approach has significantly benefitted from the publically available mouse genome sequence and the generation of unique phenotypes through random germline mutagenesis. The latter serves to expand the number of available phenotypes and allows for the genetic exploration of biological fields of interest [2], [3]. The alkylating agent *N-ethyl-N-nitrosourea* (ENU) for instance is a powerful mutagen that introduces random point mutations mostly involving A→T transversions or A→G transitions [4], [5]. With an estimate of one nucleotide change per million basepairs, ENU is believed to introduce ∼3000 genome-wide nucleotide changes translating into ∼30 heterozygous coding changes per G1 mouse and ∼4 homozygous coding changes per G3 mouse. Even so, the approach is still considered a laborious undertaking that requires significant effort in identifying causative mutations. However, recent developments in sequencing technologies (next-generation sequencing) are expected to facilitate identification of causative mutations in ENU germline mice, while at the same time significantly reducing costs.

Our laboratory applies a small scale ENU mutagenesis program to identify genetic loci required for normal lymphocyte development/function [6], [7]. Among G3 germline mutants, we identified a pedigree that contained 2 female siblings with obvious growth retardation. The phenotype was named *Lampe1* and behaved as a homozygous recessive trait. Further characterization revealed that the reduced weight coincided with the development of spontaneous microvesicular hepatosteatosis. To establish a chromosomal location, we applied low resolution mapping using a custom made 359-SNP map and analyzed 20 mice derived from intercrossed *C57BL/6J^Lampe1^*/*129SvJ* F1 hybrid mice. The critical interval for *Lampe1* resided on the distal site of chromosome 11, and contained 285 genes. Using high-density long-oligo sequence capture microarrays, we enriched all exonic sequences of the critical region including 50 bp flanking sequences and subsequently performed next-generation sequencing. A total of 3 confirmed homozygous mutations were identified with a final coverage of 96.2% of targeted DNA. One mutation represented a splice donor site mutation within the Palmitoyl Acetyl-coenzyme A oxygenase-1 (*Acox-1*) gene. Acox1 is expressed in liver and kidney cells and mediates the first enzymatic step in the peroxisomal fatty acid beta-oxidation pathway catalyzing the desaturation of acyl-CoAs to 2-trans-enoyl-CoAs. Similar to previously generated *Acox1* null mice [8], [9], the microvesicular hepatosteatosis in *Acox1^Lampe1^* mice evolved into steatohepatitis, liver cell regeneration, and ultimately development of hepatocarcinoma around 1 year of age.

## Materials and Methods

### Mice and reagents

All experiments were performed according to the US National Institutes of Health guidelines and were approved by the IACUC of The Cincinnati Children's Hospital Medical Center (protocol # 8D01008). C57BL/6J and 129X1/SvJ mice were obtained from Jackson Laboratory. *Acox1^Lampe1^* germline mice were generated at CCHMC through ENU mutagenesis. All mice were housed under specific pathogen free conditions. ENU Isopac was obtained from Sigma Chemical Co. Anti-Acox1 rabbit polyclonal antibody (Santa Cruz Biotechnology) was raised against amino acids 436–571 of ACOX1 of human origin and cross-reacted with mouse Acox1. All primers were obtained from Eurofin MWG Operon. Primers for genotyping of *Lampe1* mutants were: forward AGATTCAAGACAGAGCCGTGCAAG and reverse ACAGAGCCAAGGGTCACATCC. For cDNA amplification, forward primer TTCTCACAGCAGTGGGATTCC and reverse primer GGGCTTCAAGTGCTTGTAGTAAG were used.

### ENU mutagenesis and tissue phenotyping

Six-week-old C57BL/6J male mice were treated with ENU and subsequently bred to generate G3 germline mice as described before [10]. Six-week-old G3 animals were analyzed for developmental/immunological abnormalities and abnormalities in weight or growth rate. Liver tissue was collected and immediately fixed in 10% buffered formalin solution overnight, followed by routine paraffin embedding. Hematoxylin and eosin staining was performed on 5 µm sections from the paraffin-embedded tissue blocks for conventional light microscopy analysis. Selected paraffin blocks of liver tumors were stained for reticulin (modified Gordon and Sweet method [11], Ventana Medical Systems, Tucson, Arizona) to assess the integrity of the supporting reticulin framework of hepatocytes. The remainder of the liver tissue was snap frozen in liquid nitrogen and stored at −80°C for future analyses. Morphologic classification of the liver lesions was performed according to the International Working Party Classification of nodular lesions of the liver [12].

### SNP genotyping and low resolution whole genome mapping

To establish a critical region for the *Lampe1* mutation, we performed low resolution mapping using a custom designed genome-wide SNP map with 359 markers completely informative for C57BL/6J and 129X1/SvJ genetic backgrounds. Genotyping was performed using the Illumina GoldenGate Assay [13]. Briefly, a homozygous male *Lampe1* mutant was outcrossed to 129X1/SvJ females. Subsequent hybrid males and females were intercrossed and 20 offspring were evaluated for concordance between presence of the phenotype and homozygosity for a SNP marker, and absence of the phenotype and heterozygosity/homozygosity for the 129X1/SvJ allele. The LOD (Log Odds Distance) score (Ζ) was calculated for each marker, and used as an objective index of linkage.

### Genomic DNA capture and deep sequencing

Following the establishment of a critical region, a custom 385 K Roche-NimbleGen SeqCap array was designed to enrich exons, including 50 bp upstream/downstream sequence, defined in the *Mus musculus* NCBI build 37.1 annotation (the region of interest; Figure S2a). The Nimblegen probe selection algorithm was able to target 98.7% of the region of interest. Areas that could not be targeted were low copy repeats mostly representing non-coding bases.

Mouse genomic DNA extracted from tail clips was sonicated and adapters ligated to the fragment ends to prepare a template library for next-generation sequencing. The library was hybridized to the custom designed NimbleGen SeqCap arrays for 72 h before washing away the fragments non-specific to the target area. The enriched DNA was subsequently sequenced on an Illumina Genome Analyzer IIx using the paired-end protocol and collecting 40 bases from each read. Read alignment against the mouse genome was performed using the CASAVA software from Illumina. For variant identification a post-alignment software program was developed, designated SeqMate (Putnam and Keddache, unpublished results), to perform read sequence visualization and analysis. Specifically, this program combines the aligned reads with the reference sequence derived from the NCBI build 37.1 database and computes a distribution of call quality at each aligned base position. The variants are reported based on a number of parameters: depth of coverage, proportion of each base at a given position, and the number of different reads showing a sequence variation. For the array enrichment of *Lampe1* exons, parameters were set to: 10× minimum coverage, ignore all observations with a base quality less than or equal to 25, allow heterozygotes allelic ratios from 50%/50% to 75%/25% and a minimum of 3 read relative locations per base must be observed. Ultimately, identified mutations in the *Lampe1* critical region were confirmed by PCR amplification and Sanger sequencing using an Applied Biosystems 3730xl DNA Analyzer.

### Immunoblotting and immunohistochemistry

Immunoblotting was performed using total liver lysate from *Lampe1* or wildtype C57BL/6J mice. Cell lysis and immunoblotting were performed as described previously [14]. Double immunostaining with Kupffer cell marker anti-CD68 (Serotec, Raleigh, NC) and fat stain Oil-red O were performed in frozen liver tissue to evaluate the presence of lipid droplets within Kupffer cells using the automated Ventana immunostainer, according to manufacturer's recommendation. Appropriate positive and negative controls were used.

### Electron microscopy

Liver tissue of wildtype, heterozygote and homozygote *Lampe1* mutants (n = 3 animals per group) were fixed overnight by immersion in cold 3% glutaraldehyde solution prepared in 0.175 M cacodylate buffer followed by brief buffer rinse and post-fixation with osmium tetroxide for 1–2 hours. The fixed livers were then processed into LX-112 resin by routine techniques, stained with 2% (vol/vol) uranyl acetate and lead citrate and examined on Hitachi 7600 transmission electron microscope.

### Statistical analysis

Data were analyzed using the GraphPad Prism4® software (GraphPad Software, San Diego, CA). The statistical significance of the differences among groups was determined from the mean and standard deviation by Student's two-tailed test or by ANOVA followed by Dunnett's test for three or more groups or as indicated in the text. All data were considered statistically significant if P values were <0.05.

## Results

### Lampe1: an ENU germline mutant exhibiting growth retardation and abnormal liver development

**Figure 1:**
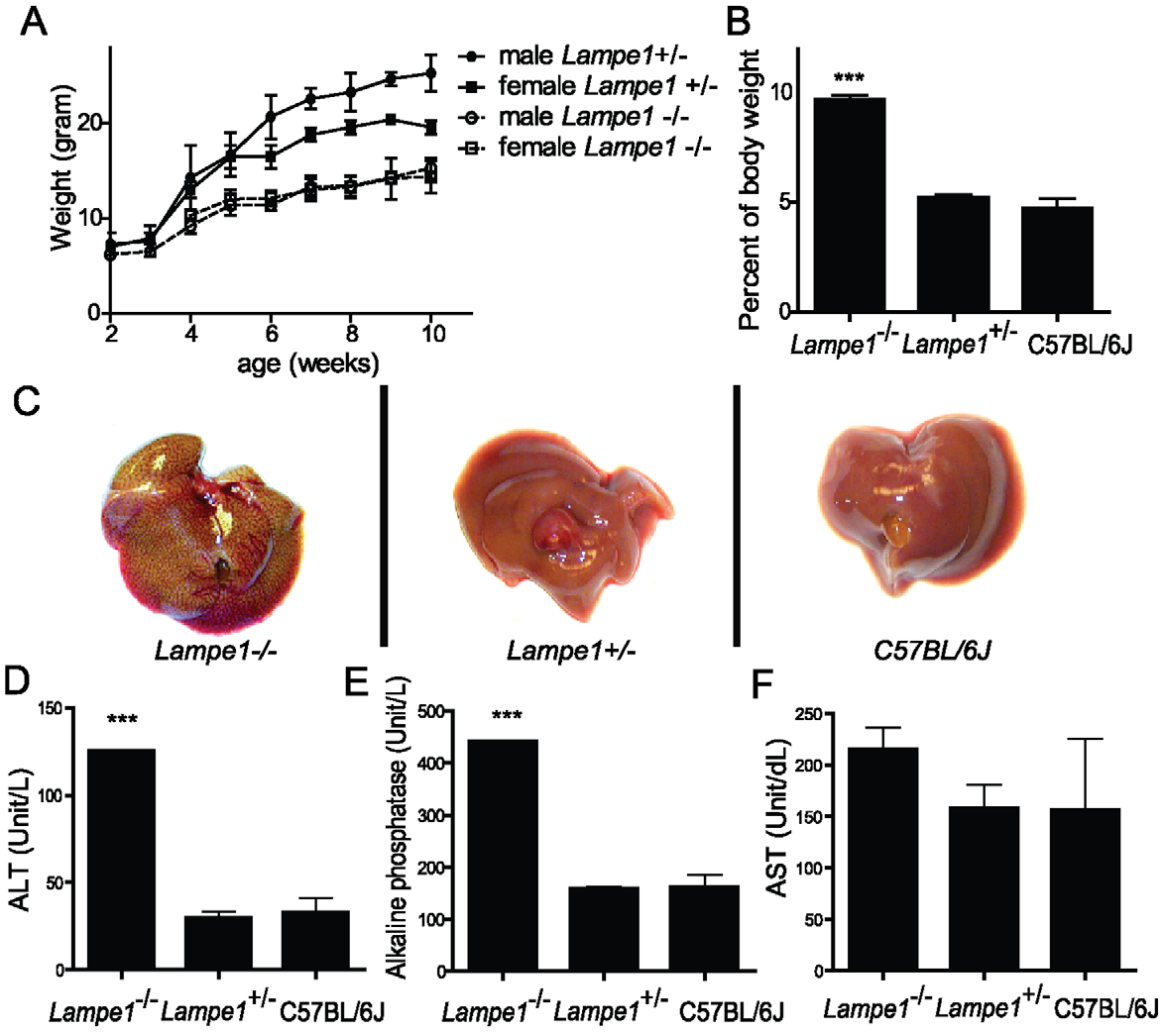
Identification and characterization of *Lampe1* ENU germline mutants. (**a**) Reduced growth of homozygote *Lampe1* male and female mice compared to heterozygote littermates. (**b**) Relative liver weight in homozygote/heterozygote *Lampe1* mutants and wildtype C57BL/6J mice. (**c**) Macroscopic view of livers from 10-week-old homozygote/heterozygote *Lampe1* mutants and C57BL/6J wildtype mice. (**d–f**) Blood levels of ALT, AP and AST liver enzymes measured in 10-week-old *Lampe1* homozygote/heterozygote mutants and C57BL/6J mice. All experiments were performed using at least n = 3 per group. *** = P<0.001.

Among G3 germline mutants, we identified two 6-week-old mice from a single pedigree that exhibited reduced weight compared to their littermates. Both were females and weighed around 11 to 12 grams, approximately 60–70 percent of normal weight for 6-week-old C57BL/6J females (Figure 1a). The G1 pedigree was selected for further breeding to establish a colony for in depth phenotype characterization and genetic analysis. The mutation was designated *Lampe1* and behaved as a strictly recessive trait. Necropsy of 10-week-old *Lampe1* mice revealed a relatively large liver compared to overall body weight (Figure 1b) and livers were macroscopically defined by yellow discoloration (Figure 1c). No other gross abnormalities were observed in *Lampe1* mutants following initial examination. Analysis of blood enzyme levels revealed significantly increased alanine transaminase and alkaline phosphatase and a trend for increased aspartate aminotransferase levels—indicating *Lampe1* mice suffer from liver injury (Figure 1d–f). No significant changes in the levels of total protein, globulin or albumin were observed between C57BL/6J and *Lampe1* heterozygous or homozygous mice (Figure S1a–c). Homozygous *Lampe1* mice exhibited a reduced fertility and were maintained as heterozygous breeders.

### Identification of the Lampe1 mutation

**Figure 2:**
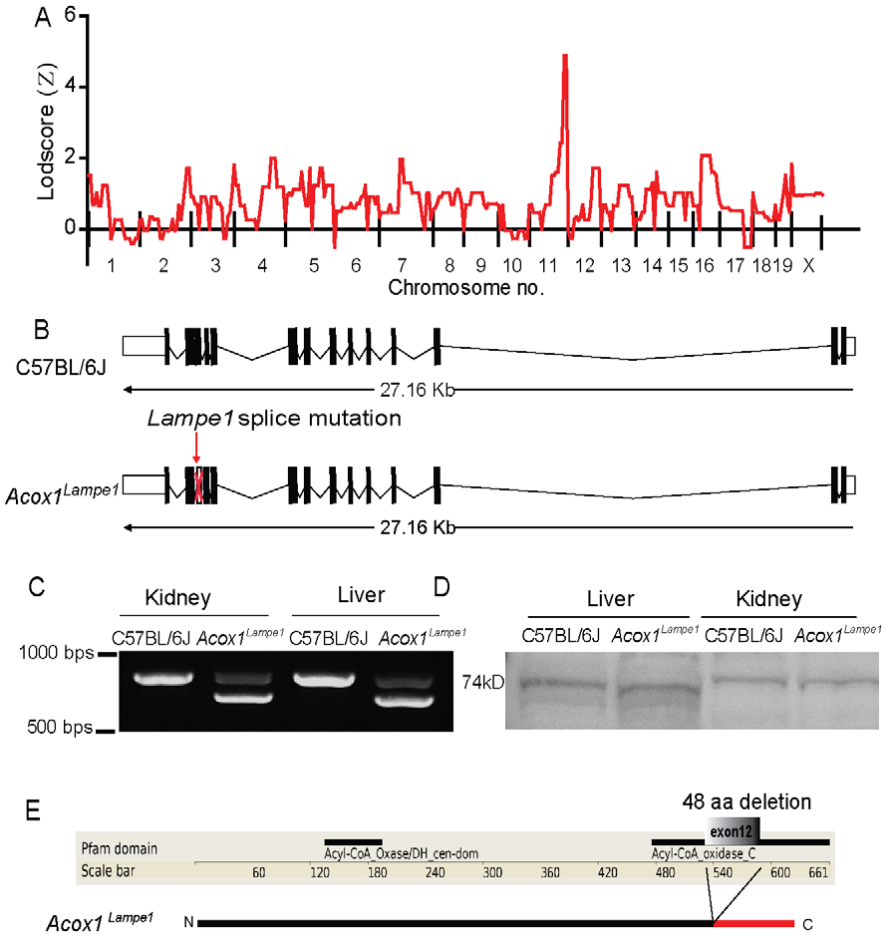
The *Lampe1* phenotype is caused by a splice site mutation in *Acox1* resulting in deletion of 48 amino-acids from the C-terminal oxidase domain. (**a**) Low-resolution mapping of the *Lampe1* mutation based on twenty mice and a panel of 359 SNPs covering the entire genome. The *Lampe1* phenotype was linked to the distal site of chromosome 11. (**b,c**) The A→C splice site mutation results in skipping of exon 12 thereby removing 144 nucleotides (**b**) as determined by amplification and sequencing of the cDNA region covering exon 9–14 (**c**). (**d,e**) At the protein level, the mutation causes the deletion of 48 amino-acids from the C-terminal acyl-CoA oxidase C domain (**e**) resulting in a slightly reduced molecular weight (69.3 kDa instead of a 74.6 kDa protein) (**d**). Similar differences in molecular weight were observed for liver and kidney tissue.

To identify the causative mutation in *Lampe1* mice we applied a 3 step approach consisting of 1) low resolution mapping, 2) exonic sequence capture and 3) next-generation sequencing of the enriched DNA. For low resolution mapping, a homozygous *Lampe1* male was outcrossed to 129X1/SvJ females and subsequently F1 hybrid offspring was intercrossed to generate F2 offspring. A total of 20 F2 offspring (5 *Lampe1* mutant- and 15 wildtype-phenotypes) were analyzed for both phenotype and genotype. Genotyping was performed using a custom-made 359-SNP map distinguishing C57BL/6J and 129X1/SvJ genetic backgrounds. Coarse mapping revealed a single peak with a LOD score of ∼5.44 for SNPs rs3699056 and rs13481247 located on the distal end of chromosome 11 (Figure 2a). The entire critical region consisted of ∼14.7 Mb genomic DNA containing 285 annotated genes. The region was defined by proximal SNP rs6386362 all the way to the distal end of chromosome 11. Using the UCSC genome browser and the NCBI build 37.1 database (*mus musculus*), the genomic coordinates for the region-specific coding sequences including an additional 50 bp flanking sequence on both sides of the exons (region of interest; Figure S2a) were defined and subsequently submitted to NimbleGen for the design of a capture array. This resulted in the targeting of 2091 exons covering 690.7 kb of total genomic DNA, of which 98.7 percent was covered by sequence capture probes (Figure S2a). The enriched genomic DNA was submitted for next-generation sequencing and 98.1% of the targeted sequence was covered with a depth of 10 reads or more. Comparison of the sequence with the reference sequence derived from the NCBI build 37.1 database identified a total of 6 homozygous and 2 heterozygous nucleotide changes within the targeted sequence (Table 1). Subsequently, each nucleotide variant was amplified by PCR from both C57BL/6 and *Lampe1* genomic DNA and analyzed by conventional Sanger sequencing. Of the identified nucleotide changes, 3 homozygote mutations were confirmed (Table 1) and 5 nucleotide changes were deemed false positives, potentially introduced during the amplification step following DNA capture. Two of the confirmed homozygous mutations represented intronic mutations, whereas one mutation represented a potential splice donor site of the Palmitoyl Acetyl-coenzyme A oxygenase-1 (*Acox1*) gene between exon 12 and 13 (Table 1 and Figure S2b).

**Table 1:** Mutations identified in the *Lampe1* critical region.

Gene affected	Relative Position	Position chrom. 11	Type of mutation	Depth*	Ref*	Confirmed	POI* observation
Dnaic2	472	114605790	intron	460	A	no	G (100)
Dnaic2	473	114605791	intron	370	G	no	A (100)
Sumo2	295	115395864	intron	216	A	no	A (72)/C (27)
Acox1	633	116035943	splice site	179	A	yes	G (100)
Evpl	849	116086932	intron	58	A	no	C (52)/A (47)
Gaa	1287	119142715	intron	38	T	yes	A (100)
Hgf	582	120333206	intron	421	G	no	A (100)
Hgf	1656	120339613	intron	824	T	yes	C (99)

*Depth: the number of reads for the specified nucleotide; Ref: reference nucleotide as annotated in the NCBI build 37.1 database; POI, Point Of Interest.

The phenotype of Acox1-deficient mice has previously been reported [8] and the *Acox1* gene represented an obvious candidate gene within the critical region. It is expressed in liver and kidney cells and is responsible for the first enzymatic step in the fatty acid beta-oxidation pathway; it catalyzes the desaturation of acyl-CoAs to 2-trans-enoyl-CoAs. The gene consists of 14 exons and disruption of the splice donor site within intron 12–13 could either lead to inclusion of the 131 bp intron in mature *Acox1* mRNA, cause the formation of a new cryptic splice site and/or result in exon skipping (Figure 2b). We therefore assessed mRNA expression and amplified cDNA generated from C57BL/6J and *Lampe1* liver tissue using primers covering exons 9–14. Gel analysis revealed a major band with a ∼150 bp reduction in size compared to the wildtype C57BL/6J amplicon in both liver and kidney tissue (Figure 2c). Subsequent sequencing of cDNA products revealed deletion of 144 nucleotides from the cDNA amplicon representing exon 12 of Acox1 (Figure 2b and Figure S2c). Skipping of exon 12 would cause the deletion of 48 amino acids from the Acyl-CoA oxidase domain at the c-terminal part of Acox1 (Figure 2e). In addition, a second band of ∼780 bps suggested an alternative splice form was expressed in the liver and kidney of *Lampe1* mice. Subsequent isolation and sequencing of this amplicon revealed the creation of new splice site within intron 12–13, resulting in the inclusion of 25 bps of intronic sequence in the *Lampe1* transcript (Figure S2c). Translation of this transcript is predicted to cause a frameshift and alternative coding at amino acid 577 followed by a premature stop at amino acid 618 (Figure S2d), thus resulting in partial deletion of the C-terminal Acyl-CoA oxidase domain. Immunoblot analysis of Acox1 protein expression in liver cells or kidney tissue revealed a slightly reduced molecular weight of the Acox1 protein in *Lampe1* mutant cells, consistent with a 48 amino acid deletion (Figure 2d). Based on the observations, we concluded that the identified variant causing disruption of the *Acox1* splice donor site was responsible for the development of hepatosteatosis and growth retardation in *Lampe1* mice (from here on designated *Acox1^lampe1^* mice).

### Hepatic phenotype of Acox1^lampe1^ mice

**Figure 3:**
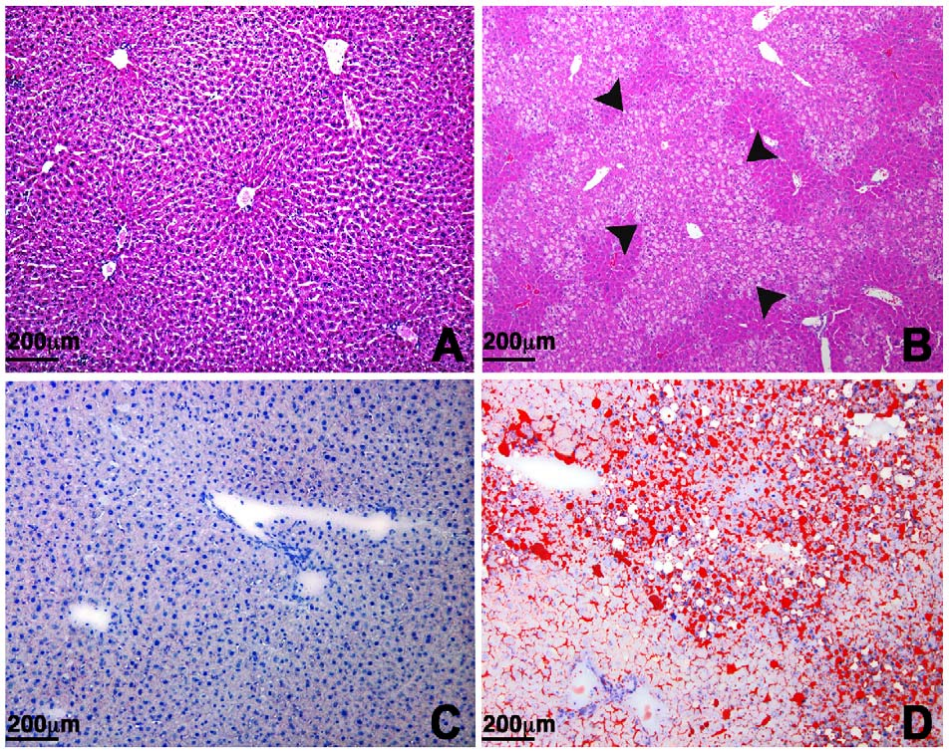
Histopathology of the liver. (**a,b**) Hematoxylin and eosin staining of control (**a**) and *Acox1^lampe1^*-mutant (**b**) liver at 2 months showing severe microvesicular steatosis affecting pericentral and midzone hepatocytes (arrows) with relative sparing of periportal hepatocytes. (**c,d**) Oil-red O staining reveals fat accumulation in a similar zonal distribution in *Acox1^lampe1^* livers (**d**), while no staining is observed in livers from control mice (**c**). (**a–d**: 100× magnification).

**Figure 4:**
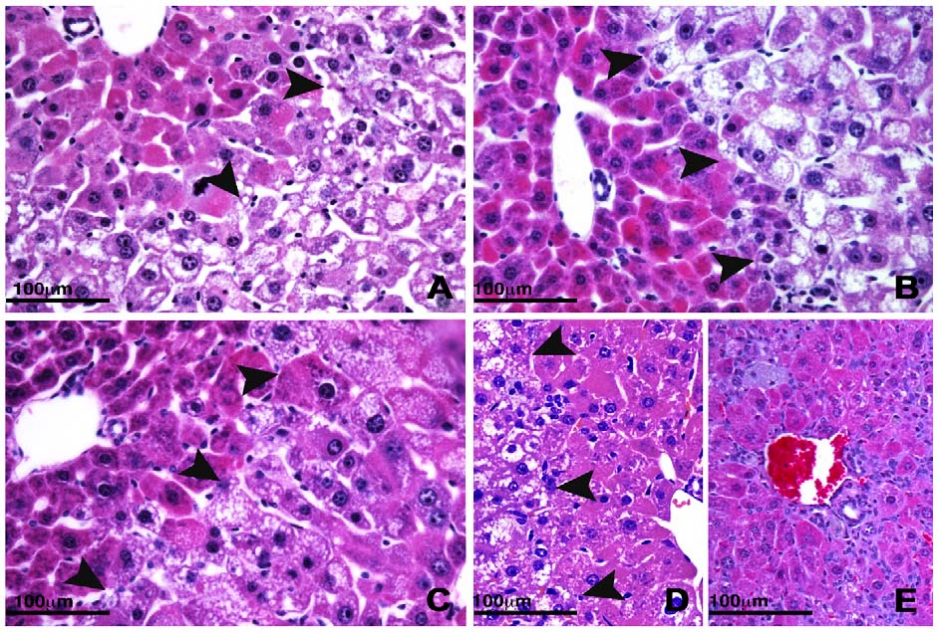
Persistent liver injury in *Acox1^lampe1^* mice. (**a–c**) A zonal pattern of steatosis (arrows) with sparing of periportal hepatocytes is observed in *Acox1^lampe1^* livers at 2–6 months of age. At 6 months, the distribution of fat is highly heterogeneous with some residual areas of steatosis (**d**; arrows), admixed with large zones that are devoid of fat and predominantly composed of hepatocytes with deeply eosinophilic cytoplasm(**e**). (**a**), 2 months; (**b**), 3 months; (**c**), 4 months; (**d**) and (**e**), 6 months. Hematoxylin and eosin; 400× magnification.

**Figure 5:**
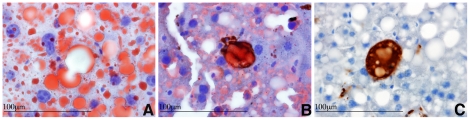
Lipid accumulation in Kupffer cells of *Acox1^lampe1^* mice. (**a**) Oil-red O stain demonstrates the presence of intracytoplasmic lipid vacuoles in hepatocytes and Kupffer cells. (**b**) Double immunostaining with Oil-Red O and anti-CD68 confirms the location of the fat droplets in the cytoplasm of sinusoidal Kupffer cells. (**c**) CD68 highlights an enlarged Kupffer cell with abundant vacuolated cytoplasm. Magnification 1000×.

To examine the *Acox1^lampe1^* phenotype in more detail, we performed a thorough analysis of the liver histology and ultrastructure at various ages. At 8 weeks, the liver of *Acox1^lampe1^* mice showed extensive microvesicular steatosis involving most of the hepatic lobule with sparing of periportal hepatocytes (Figure 3b–d). This striking zonal pattern of steatosis persisted at 2, 3 and 4 month-old mice (Figure 4a–c). At 6 months, the liver parenchyma contained areas with steatotic hepatocytes (Figure 4d) as well as multiple lobules showing complete resolution of steatosis (Figure 4e), except for the presence of distended Kupffer cells containing lipid material (Figure 4e). Double immunostaining with anti-CD68 antibody and Oil Red O performed on frozen liver samples confirmed the presence of fat droplets within the sinusoidal Kupffer cells (Figure 5a–c).

**Figure 6:**
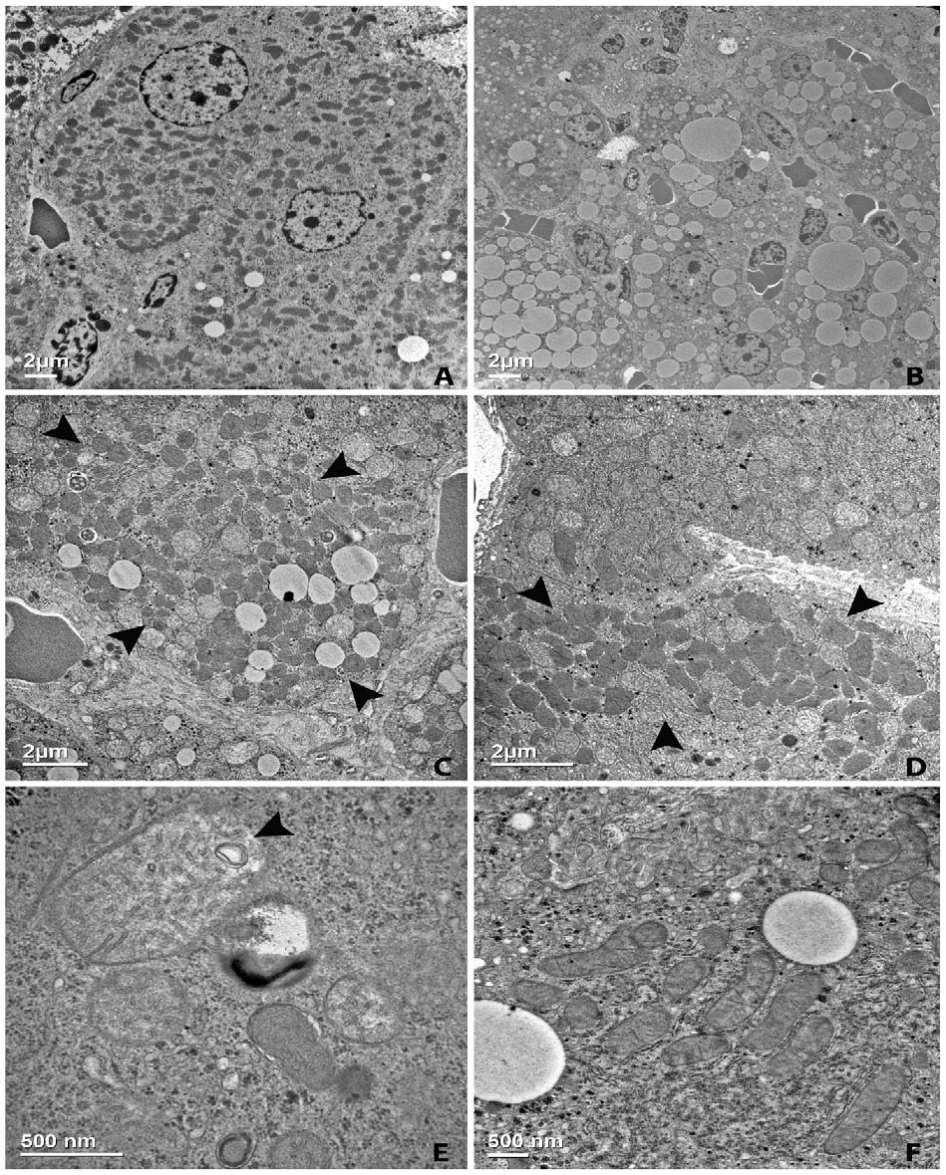
Ultrastructural changes in *Acox1^lampe1^* livers. Electron micrograph from the liver of an 8-week-old *Acox1^lampe1^* mouse (**b–e**) compared to age-matched control (**a, f**). The appearance of normal hepatocytes from an 8-week-old wild-type mouse (**a**) contrasts with hepatocytes from *Acox1^lampe1^* liver, which show small and large lipid droplets displacing the cytoplasmic organelles (**b**). Increased number of peroxisomes in hepatocytes immediately adjacent to the steatosis, often arranged in clusters (**c**, arrows). In areas of the parenchyma away from the fat, only occasional clusters are observed in periportally located hepatocytes in a patchy distribution (**d**, arrows). The mitochondria in *Acox1^lampe1^* hepatocytes show marked enlargement with swelling and decreased matrix density and presence of laminated material (**e**, arrow) compared to control mice (**f**).

Ultrastructurally, *Acox1^lampe1^* livers revealed numerous lipid vacuoles of varying sizes within the cytoplasm of zones 2 and 3 hepatocytes, displacing most of the cytoplasmic organelles (Figure 6b). The steatotic hepatocytes either lacked or showed diminished numbers of peroxisomes. In contrast, the hepatocytes immediately adjacent to the fatty area demonstrated a relatively higher amount of peroxisomes, which formed discrete clusters in the cytoplasm (Figure 6c). Away from the fatty area, the distribution of peroxisomes was heterogeneous, with patchy clusters observed in some of the hepatocytes around zone 1 (Figure 6d). The mitochondria were swollen with a markedly hypodense matrix. Laminated material (Figure 6e) and lipid vacuoles were seen within the mitochondrial membranes or matrix.

**Figure 7:**
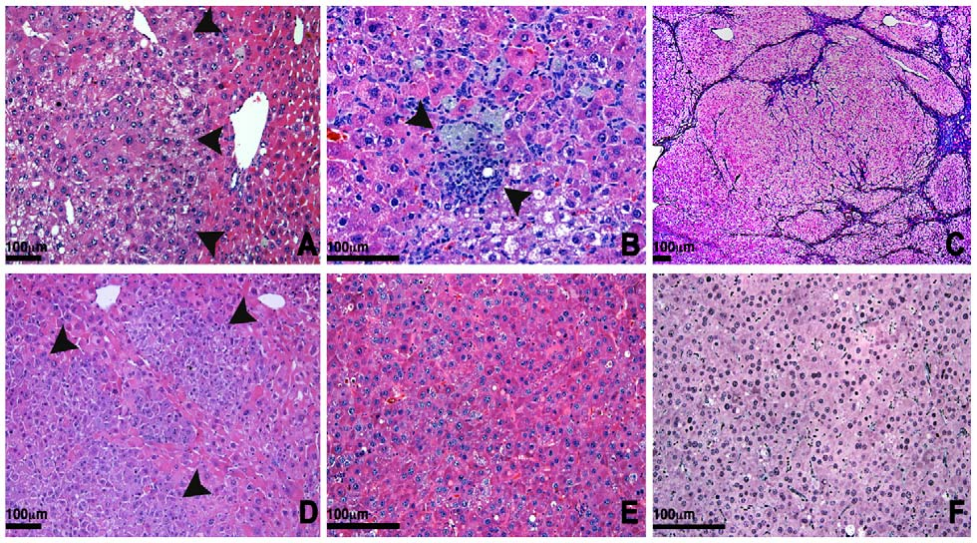
Evolution of steatosis to steatohepatitis and neoplasia. (**a**) Histology of the liver of a 4 month-old *Acox1^lampe1^* mouse showing fat accumulation in a zonal distribution around the central veins (magnification 200×) and (**b**) scattered foci of lobular inflammation with aggregates of macrophages at 4 months (magnification 400×). (**c**) At 12 months, the liver architecture is markedly disrupted by expansile nodules replacing most of the parenchyma (magnification 200×). (**d**) Foci of dysplastic hepatocytes and dysplastic nodules were present throughout the liver and some of the nodules contained areas of frank hepatocellular carcinoma (**e**), with loss of reticulin fibers (**f**). Hematoxylin and eosin (**a–e**) and reticulin (**f**) stain. 6D–F 400× magnifications.

The resolution of poor weight gain observed in young *Acox1^lampe1^* mice raised the possibility that the liver injury resolved. To address this possibility, we performed a detailed histological survey of the mouse liver well into adulthood. We found partial resolution of the hepatic steatosis (Figures 4e and 7a), accompanied by lobular inflammatory infiltrates, comprised of numerous clusters of lymphocytes and Kupffer cells (Figure 7b). At necropsy, the livers of 15 month-old mice were significantly enlarged and contained distinct nodular lesions with slightly different color from the surrounding hepatic parenchyma. On histology, the lobular architecture was disrupted by almost complete replacement of the parenchyma by nodules of varying sizes (Figure 7c). The nodules consisted of eosinophilic and/or basophilic hepatocytes with focal or diffuse dysplastic changes and increased cell density (Figure 7d). Microscopic foci of large and small cell change were scattered throughout the parenchyma. Histological features of hepatocellular carcinoma (HCC) such as increased cellularity, liver cell plate thickening and expansile or invasive growth patterns occurred in multiple areas and intermingled indiscernibly with the dysplastic foci (Figures 7e). HCC showed solid, trabecular and sinusoidal patterns and focal to complete loss of the reticulin network supporting the diagnosis of malignant transformation (Figure 7f).

Overall, *Acox1^lampe1^* mice present a similar phenotype as previously described for *Acox1* knockout mice [8] and exhibit hepatosteatosis at an early stage that slowly evolves into hepatosteatitis and induction of liver cell regeneration, ultimately driving the development of hepatocarcinoma around 1-year of age.

## Discussion

The rate-limiting step in ENU mutagenesis has long been the identification of causative mutations. Here we report a cost-effective approach to identify phenotype-related nucleotide variants. Specifically, we targeted the *Lampe1* locus—an ENU germline mutant exhibiting growth retardation and spontaneous hepatosteatosis, evolving to steatohepatitis and hepatocellular carcinoma. We performed low resolution mapping based on a limited number of meioses resulting in the identification of a ∼14 Mb critical region. Subsequently we applied targeted exon enrichment—with the inclusion of 50 bp proximal/distal flanking sequence for all exons—and analyzed the enriched DNA using next-generation sequencing technology. The approach effectively identified a donor splice site mutation in the Palmitoyl Acetyl-coenzyme A oxygenase-1 (*Acox1*) gene that was responsible for the described phenotype in *Lampe1* mice. Importantly, this approach eliminated the necessity for fine-mapping—a laborious, time consuming and relative expensive process related to forward genetics and ENU mutagenesis. The coverage of targeted DNA for the *Lampe1* critical region was exceptional, with 98.1% of the targeted region being sequenced with a minimal depth of 10. This exceptionally high coverage was predominantly due to the fact that a relatively small genomic interval was targeted (690.7 kb of targeted DNA). The Nimblegen array used in this study has 384,000 probes and a capacity to capture up to 5 Mb of DNA sequence. This means that the tiling across the target region was approximately 1 probe every 1 or 2 bases on average. This optimal capture design, combined with the large number of reads generated (average coverage depth of 453× over the target region) were critical elements that allowed us to obtain 10× coverage on 98.1% of the target region.

Although the size and gene density of a critical region may vary somewhat between ENU germline mutants, we anticipate that the obtained sequence coverage can generally be reproduced with a similar probe design and total number of reads generated. In addition, the enrichment capacity of 2.1 M arrays captures ∼30 Mb of targeted sequence, allowing for the analysis of the entire mouse exome. Nonetheless, causality (i.e. through linkage analysis or genetic confirmation) will remain an essential aspect of forward genetics.

The effective identification of genomic nucleotide changes also expands the possibilities in (immune)phenotyping. In many instances, phenotypes are lost or significantly influenced by modifier loci located on outcross strains carrying a high degree of genetic variation. For example, the genetic dissection of NK cell function has proven challenging in our laboratory because of the large variation in NK cell ligands/receptors existing on different mouse backgrounds (Hoebe, unpublished results). The current approach avoids fine mapping and allows for the exploration of phenotypes that are more subtle and can be traced following the outcross to strains with minimal genetic variation (e.g. the C57BL/6J and C57BL/10J strains). Thus the ability to effectively target large genomic intervals not only facilitates mutation discovery, but also expands the number of phenotypes that can be explored. Interestingly, ENU has been described as a powerful mutagen that at optimal doses can induce about one base-pair change per million base-pairs of genomic DNA [15]. Here we identified and confirmed 3 base-pair changes in 690 kb of sequenced DNA, suggesting a higher mutation frequency induced by ENU than previously estimated. Further analysis of large areas of genomic DNA using next-generation sequencing should ultimately provide a better idea of the exact mutation frequency for ENU in mice.

The pathogenesis observed in *Acox1^lampe1^* mice largely resembles the pathogenesis observed in *Acox1* null mice as previously published by Reddy's group [8], [9], [16]. The latter presented growth retardation, severe microvesicular hepatic steatosis and sustained activation of peroxisome proliferator-activated receptor-α (PPARα) [8], [9], [16]. Acox1 is expressed as a 72 kDa precursor protein that enters the peroxisome via the C-terminal Ser-Lys-Leu-related tripeptide target signal [17]. Within the peroxisome, Acox1 is cleaved by the protease trypsin domain-containing 1 (Tysdn1), resulting into a 50 kDa N-terminal and a 22 kDa C-terminal subunit ultimately generating heterodimeric complexes comprised of all three subunits [17], [18]. The *Lampe1* mutation affects the C-terminal domain of Acox1 containing the Acetyl-CoA oxidase domain—an essential domain for enzyme activity. The 48 amino acid deletion, however, does not affect the C-terminal signal peptide (SKL) nor does it affect the Tysdn1-specific cleavage site, suggesting normal localization and processing of the mutant Acox1 protein in the peroxisome may still occur. Indeed, immunoblot analysis reveals a quantitative similar expression of Acox1, albeit with a reduced molecular weight in liver and kidney of *Acox1^lampe1^* mice. Although we have not performed enzymatic activity assays to confirm the loss of Acox1 function in Lampe1 mutant mice, the 48 amino-acid deletion from the Acetyl-CoA oxidase domain is likely to render the protein functionally deficient and cause a phenotype similar to the previously reported *Acox1* null mice. Nonetheless, subtle differences seem apparent between both mouse models. First of all, the distribution of microvesicular fatty metamorphosis in 2-month-old *Acox1^lampe1^* livers is zonal and observed in zones 2 and 3 but not zone 1, whereas Fan et al reported fatty changes in all hepatocytes irrespective of their distribution in the liver of *Acox1^−/−^* mice at similar age [8], [9]. Notably, we observed a more severe and uniform fatty metamorphosis in the liver following the outcross of the *Lampe1* mutation onto 129X1/SvJ background (results not shown), suggesting that modifier loci on the 129X1/SvJ or C57BL/6J to some extent influence the degree of hepatosteatosis.

Acox1 is essential for β-oxidation of very-long chain fatty acyl-CoA and arachidonic acid metabolites such as 8(S)-hydroxy-eicosatetraenoic acid and leukotriene B_4_ (LTB_4_). In the absence of functional Acox1, these lipid metabolites accumulate and act as natural ligands for the nuclear receptor PPARα [19], [20], [21], initiating a unique transcriptional program following heterodimeric association of PPARα with nuclear retinoid X receptor (RXR) [22]. Ultimately, this complex induces a select set of genes encoding the peroxisomal β-oxidation system, including Acox1 but also enzymes such as cytochrome P450 4A isoforms (CYP4A1 and CYP4A3) [23], [24]. The latter metabolize long-chain fatty acids via the ω-oxidation pathway. In the absence of functional Acox1, this pathway becomes an important metabolic pathway for long-chain fatty acids, resulting in accumulation of toxic dicarboxilic acids (DCAs) causing mitochondrial damage [21], [25]. In addition, the dramatic increase in enzyme expression related to lipid oxidation coincides with high levels of reactive oxygen species such as hydrogen peroxide, ultimately causing cell death, regenerative cell proliferation and hepatocarcinogenesis [21]. Indeed, compound homozygous *PPARα^−/−^*, *Acox1^−/−^* mice lack increased peroxisomal proliferation and exhibit reduced steatosis and liver cell proliferation [26], thus verifying the essential role of PPARα in the pathogenesis of *Acox1*-deficient mice. Although in our *Acox1^lampe1^* model we have not directly measured the activation of PPARα and expression of CYP450 enzymes, the morphologic changes in the liver of *Acox1^lampe1^* mice were similar to the previously published *Acox1*-null mice by Reddy's group. Finally, the increased number of peroxisomes in hepatocytes was a common feature in both *Acox1^−/−^* and *Acox1^lampe1^* mice. In addition, stress changes in the mitochondria were prevalent in *Acox1^lampe1^* mice.

Recent epidemiological reports suggest that obesity significantly increases the risk of hepatocellular carcinoma in humans [27], [28]. Obesity is associated with simple steatosis (60%), nonalcoholic steatohepatitis (25%) whereas 3% to 5% of obese people exhibit liver cirrhosis [29]. Steatosis is associated with increased levels of ROS and the induction of low level inflammatory response including the release of proinflammatory cytokines such TNFα [29]. A report by Park et al suggests that low-level inflammation, specifically the activation of STAT3 via cytokines such as IL-6, is a requirement for development of hepatocellular carcinoma in mouse models with dietary or genetic obesity [30]. Currently, the molecular basis for (sterile) low level inflammation in Acox1-deficient mice or for that matter in the context of obesity is poorly defined. An important trigger of inflammation may be derived from the excessive cell death—possibly due to CYP4A-mediated ROS production and DCA-release—observed in *Acox1*-deficient mice. This may induce further release of lipids and other components sensed by innate immune receptors ultimately causing steatohepatitis, cell proliferation and hepatocellular carcinoma. A large number of studies have reported the induction of proinflammatory cytokines following exposure of dendritic cells or macrophages to cell death [31], [32], [33], [34]. The regulatory pathways and innate receptors involved in mediating inflammation via cell death have been poorly defined particularly in the context of dying cells with excessive lipid accumulation as observed in *Acox1*-deficient mice. Thus *Acox1^lampe1^* mice may present a unique model to further dissect the (sterile) inflammatory pathways that drive liver regeneration and ultimately hepatocellular carcinoma.

## Supporting Information

Figure S1 Protein (a), globulin (b) and albumin (c) levels measured in blood from 10-week-old *Lampe1* homozygote/heterozygote mutants or C57BL/6J mice. (n = 3 per group).
(TIF)

Figure S2 Targeting the Lampe1 critical region for exon enrichment using high-density long-oligo arrays. (a) Exons including 50 bp upstream/downstream sequence were defined based on the *Mus musculus* NCBI build 37.1 database (primary target region) and submitted for enrichment. The Nimblegen probe selection algorithm captured 98.7% of targeted sequence with 1.3% being excluded based on low copy repeat sequence mostly representing non-coding sequence. (b) The Lampe1 mutation represents a splice donor site mutation in intron 12–13 of *Acox1*. (c) Alignment of *Acox1* cDNA from wildtype and *Acox1^lampe1^* mutant mice, for the major and minor splice forms, as determined by sequencing. (d) Predicted c-terminal protein sequence of the minor splice form of *Acox1^lampe1^*.
(TIF)

## References

[1] Beutler B, Jiang Z, Georgel P, Crozat K, Croker B (2006). Genetic analysis of host resistance: Toll-like receptor signaling and immunity at large.. Annu Rev Immunol.

[2] Beutler B (2004). Inferences, questions and possibilities in Toll-like receptor signalling.. Nature.

[3] Beutler B, Du X, Xia Y (2007). Precis on forward genetics in mice.. NatImmunol.

[4] Justice MJ, Carpenter DA, Favor J, Neuhauser-Klaus A, Hrabe dA (2000). Effects of ENU dosage on mouse strains.. MammGenome.

[5] Kile BT, Hentges KE, Clark AT, Nakamura H, Salinger AP (2003). Functional genetic analysis of mouse chromosome 11.. Nature.

[6] Barnes MJ, Aksoylar H, Krebs P, Bourdeau T, Arnold CN (2010). Loss of T cell and B cell quiescence precedes the onset of microbial flora-dependent wasting disease and intestinal inflammation in Gimap5-deficient mice.. J Immunol.

[7] Barnes MJ, Krebs P, Harris N, Eidenschenk C, Gonzalez-Quintial R (2009). Commitment to the regulatory T cell lineage requires CARMA1 in the thymus but not in the periphery.. PLoSBiol.

[8] Fan CY, Pan J, Chu R, Lee D, Kluckman KD (1996). Hepatocellular and hepatic peroxisomal alterations in mice with a disrupted peroxisomal fatty acyl-coenzyme A oxidase gene.. J Biol Chem.

[9] Fan CY, Pan J, Chu R, Lee D, Kluckman KD (1996). Targeted disruption of the peroxisomal fatty acyl-CoA oxidase gene: generation of a mouse model of pseudoneonatal adrenoleukodystrophy.. Ann N Y Acad Sci.

[10] Hoebe K (2009). Genetic dissection of Toll-like receptor signaling using ENU mutagenesis.. Methods Mol Biol.

[11] Gordon H, Sweets HH (1936). A Simple Method for the Silver Impregnation of Reticulum.. Am J Pathol.

[12] Wanless I (1995). Terminology of nodular hepatocellular lesions. International Working Party.. Hepatology.

[13] Barker DL, Hansen MS, Faruqi AF, Giannola D, Irsula OR (2004). Two methods of whole-genome amplification enable accurate genotyping across a 2320-SNP linkage panel.. Genome Res.

[14] Kim SO, Jing Q, Hoebe K, Beutler B, Duesbery NS (2003). Sensitizing anthrax lethal toxin-resistant macrophages to lethal toxin-induced killing by tumor necrosis factor-alpha.. J Biol Chem.

[15] Concepcion D, Seburn KL, Wen G, Frankel WN, Hamilton BA (2004). Mutation rate and predicted phenotypic target sizes in ethylnitrosourea-treated mice.. Genetics.

[16] Fan CY, Pan J, Usuda N, Yeldandi AV, Rao MS (1998). Steatohepatitis, spontaneous peroxisome proliferation and liver tumors in mice lacking peroxisomal fatty acyl-CoA oxidase. Implications for peroxisome proliferator-activated receptor alpha natural ligand metabolism.. J Biol Chem.

[17] Miura S, Kasuya-Arai I, Mori H, Miyazawa S, Osumi T (1992). Carboxyl-terminal consensus Ser-Lys-Leu-related tripeptide of peroxisomal proteins functions in vitro as a minimal peroxisome-targeting signal.. J Biol Chem.

[18] Kurochkin IV, Mizuno Y, Konagaya A, Sakaki Y, Schonbach C (2007). Novel peroxisomal protease Tysnd1 processes PTS1- and PTS2-containing enzymes involved in beta-oxidation of fatty acids.. EMBO J.

[19] Devchand PR, Keller H, Peters JM, Vazquez M, Gonzalez FJ (1996). The PPARalpha-leukotriene B4 pathway to inflammation control.. Nature.

[20] Hostetler HA, Petrescu AD, Kier AB, Schroeder F (2005). Peroxisome proliferator-activated receptor alpha interacts with high affinity and is conformationally responsive to endogenous ligands.. J Biol Chem.

[21] Yeldandi AV, Rao MS, Reddy JK (2000). Hydrogen peroxide generation in peroxisome proliferator-induced oncogenesis.. Mutat Res.

[22] Kliewer SA, Umesono K, Mangelsdorf DJ, Evans RM (1992). Retinoid X receptor interacts with nuclear receptors in retinoic acid, thyroid hormone and vitamin D3 signalling.. Nature.

[23] Hardwick JP, Song BJ, Huberman E, Gonzalez FJ (1987). Isolation, complementary DNA sequence, and regulation of rat hepatic lauric acid omega-hydroxylase (cytochrome P-450LA omega). Identification of a new cytochrome P-450 gene family.. J Biol Chem.

[24] Reddy JK, Goel SK, Nemali MR, Carrino JJ, Laffler TG (1986). Transcription regulation of peroxisomal fatty acyl-CoA oxidase and enoyl-CoA hydratase/3-hydroxyacyl-CoA dehydrogenase in rat liver by peroxisome proliferators.. Proc Natl Acad Sci U S A.

[25] Rao MS, Reddy JK (2004). PPARalpha in the pathogenesis of fatty liver disease.. Hepatology.

[26] Hashimoto T, Fujita T, Usuda N, Cook W, Qi C (1999). Peroxisomal and mitochondrial fatty acid beta-oxidation in mice nullizygous for both peroxisome proliferator-activated receptor alpha and peroxisomal fatty acyl-CoA oxidase. Genotype correlation with fatty liver phenotype.. J Biol Chem.

[27] Bianchini F, Kaaks R, Vainio H (2002). Overweight, obesity, and cancer risk.. Lancet Oncol.

[28] Calle EE, Kaaks R (2004). Overweight, obesity and cancer: epidemiological evidence and proposed mechanisms.. Nat Rev Cancer.

[29] Caldwell SH, Crespo DM, Kang HS, Al-Osaimi AM (2004). Obesity and hepatocellular carcinoma.. Gastroenterology.

[30] Park EJ, Lee JH, Yu GY, He G, Ali SR (2010). Dietary and genetic obesity promote liver inflammation and tumorigenesis by enhancing IL-6 and TNF expression.. Cell.

[31] Janssen E, Tabeta K, Barnes MJ, Rutschmann S, McBride S (2006). Efficient T cell activation via a Toll-Interleukin 1 Receptor-independent pathway.. Immunity.

[32] Kono H, Chen CJ, Ontiveros F, Rock KL (2010). Uric acid promotes an acute inflammatory response to sterile cell death in mice.. J Clin Invest.

[33] Rock KL, Latz E, Ontiveros F, Kono H (2010). The sterile inflammatory response.. Annu Rev Immunol.

[34] Obeid M, Tesniere A, Ghiringhelli F, Fimia GM, Apetoh L (2007). Calreticulin exposure dictates the immunogenicity of cancer cell death.. NatMed.

