# Supplementary figures and images for: Lampe1: An ENU-Germline Mutation Causing Spontaneous Hepatosteatosis Identified through Targeted Exon-Enrichment and Next-Generation Sequencing
Source: PLoS One. 2011 Jul 7;6(7):e21979. doi: 10.1371/journal.pone.0021979 (PMC3131302; doi:10.1371/journal.pone.0021979)

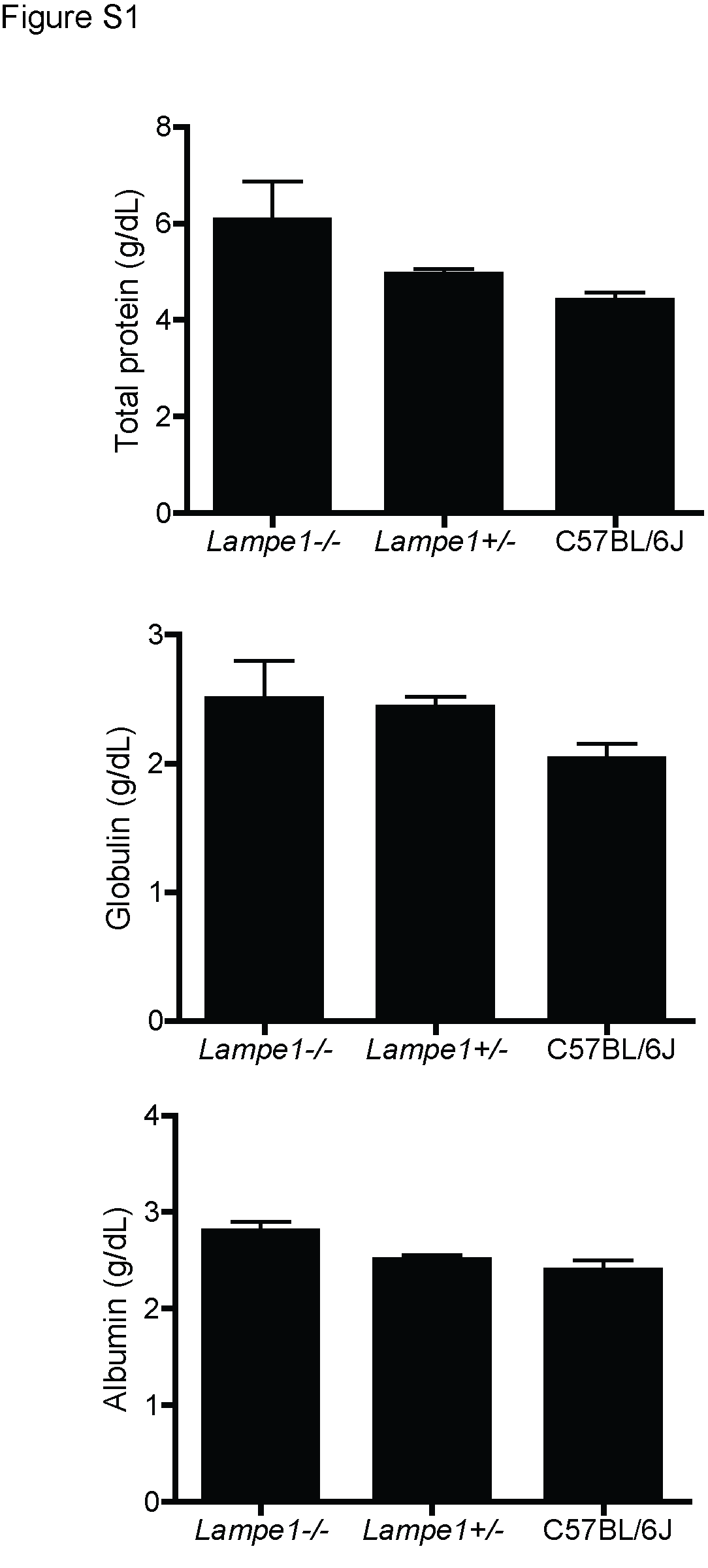

Supplement: Figure S1 — Protein (a), globulin (b) and albumin (c) levels measured in blood from 10-week-old Lampe1 homozygote/heterozygote mutants or C57BL/6J mice. (n = 3 per group). (TIF) [file pone.0021979.s001.tif]

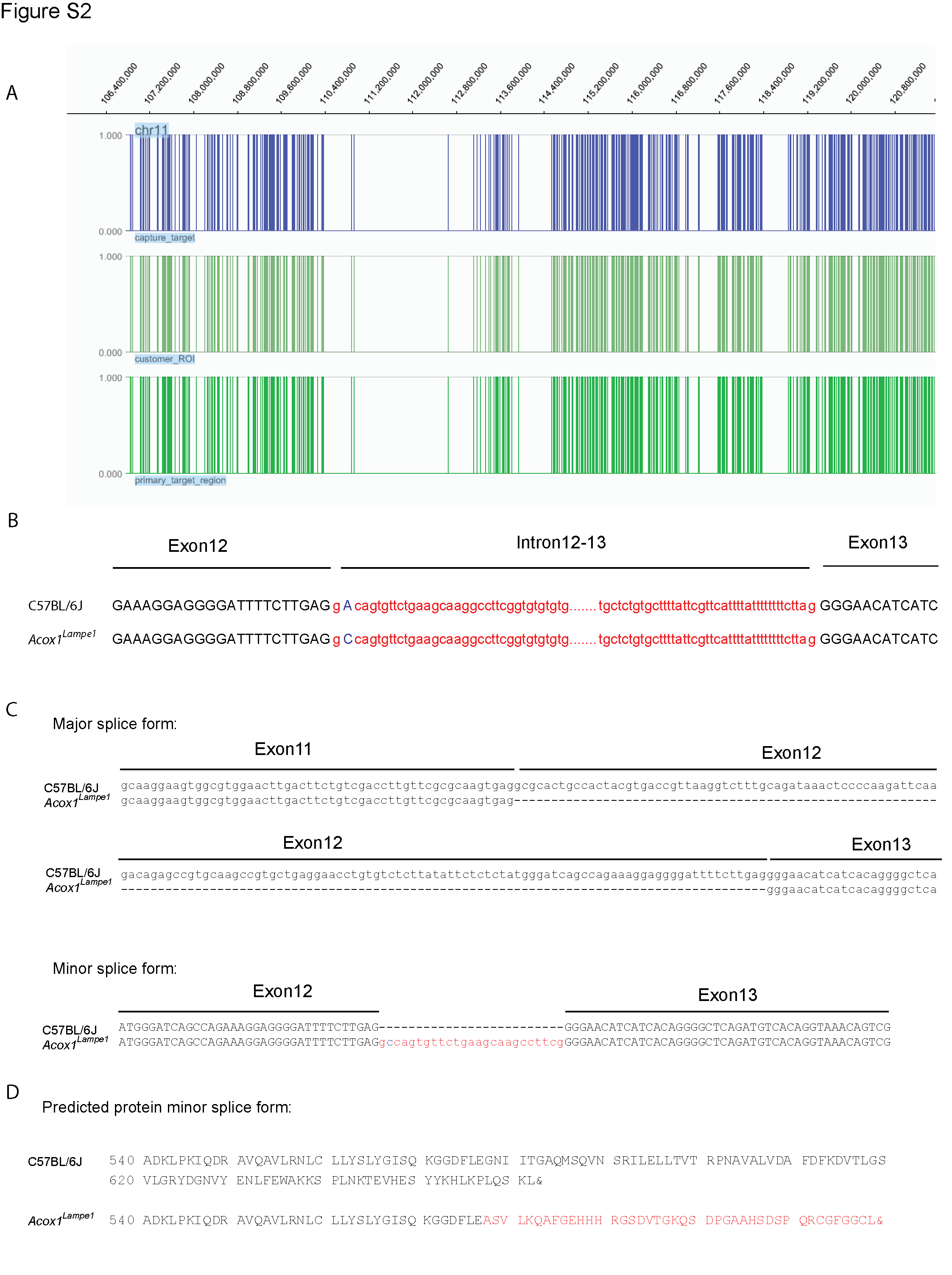

Supplement: Figure S2 — Targeting the Lampe1 critical region for exon enrichment using high-density long-oligo arrays. (a) Exons including 50 bp upstream/downstream sequence were defined based on the Mus musculus NCBI build 37.1 database (primary target region) and submitted for enrichment. The Nimblegen probe selection algorithm captured 98.7% of targeted sequence with 1.3% being excluded based on low copy repeat sequence mostly representing non-coding sequence. (b) The Lampe1 mutation represents a splice donor site mutation in intron 12–13 of Acox1. (c) Alignment of Acox1 cDNA from wildtype and Acox1lampe1 mutant mice, for the major and minor splice forms, as determined by sequencing. (d) Predicted c-terminal protein sequence of the minor splice form of Acox1lampe1. (TIF) [file pone.0021979.s002.tif]
